# The Features of Modeling Mediation in Digital Support for Formation of Multiplicative Concepts

**DOI:** 10.11621/pir.2024.0207

**Published:** 2024-06-01

**Authors:** Elena Vysotskaya, Maria Yanishevskaya, Anastasia Lobanova

**Affiliations:** a *Federal Scientific Centre of Psychological and Multidisciplinary Research, Moscow, Russia*

**Keywords:** multiplicative concepts, balance scale problem, joint activity, concept formation, computer-supported learning

## Abstract

**Background.:**

The formation of multiplicative concepts of complex structure is a challenge for educational design. Students’ typical mistakes and strategies spontaneously obtained through hands-on trials in solving balance scale problems have been at the center of many studies within this trend. However, the consideration of relevant concept-mediated actions based on Learning Activity Theory (Davydov) remains a relevant problem.

**Objective.:**

We aimed to develop a feasible framework for digital support of students’ learning actions in this domain. The productiveness of individual and joint forms of work with dynamic objects in a digital environment, mediated with conceptual modeling tools, was compared.

**Design.:**

The participants were 181 fifth-grade students (11–12 years old). The first group (123 students) was taught a special procedure of modeling, which they then could test during individual computer-supported problem-solving. The second group (58 students) worked in pairs (jointly), using the same procedure. The pre- and post-tests included challenging problems on prediction of the balance state and ways to regain equilibrium.

**Results.:**

Comparison of the pre- and post-test results of the joint computer-supported activity instruction revealed students’ progress in solving critical tasks as guided by the conceptual modeling procedure of load evaluation instead of “empirical” correlations of weights and distances. The individual computer-supported work, however, failed to overcome the belief of some students in the efficacy of trial-and-error methods as applied to the digital simulation with instant feedback.

**Conclusion.:**

The special organization of the computer-supported concept-mediated joint activity may promote multiplicative concept formation.

## Introduction

With this research we contribute to studying some important issues of introducing computers to teaching. Based on the activity approach in education (Davydov, 2008; Davydov et al., 1983; Engeness, 2021; Galperin, 1989; Leontiev, 2003; Rubtsov, 1996; Rubtsov & Ulanovskaya, 2021; Talyzina, 1988), which proved to be productive for understanding concept formation, we consider the necessary stages of concept acquisition and develop a feasible framework for digital support of students’ learning actions in this domain.

Our study regards the prerequisites of complex multiplication-based concept formation within the context of balance scale problems. This task, introduced by B. Inhelder and J. Piaget (1958), and later developed by R.S. Siegler (2013), has become a classical context to examine the structure of complex concepts and the operations behind them, and strategies for solving some particular tasks of balancing scales. Much attention has been paid to the analysis of students’ mistakes, based on flaws in logical multiplication in application to operating quantities (Kloosterman, 2010; Lamon, 2020; Siegler & Chen, 2008). Following J. Piaget, all the researchers attributed the efficacy of handling such concepts to the “age-dependent” ability of students to distinguish the two latent parameters that affect the balance, and to consider them simultaneously (Bonawitz et al., 2012; Boom et al., 2001; Howe et al., 2011; Jansen & van der Maas, 2002; Siegler, 2013; Wachsmuth et al., 1983). The specifics of cooperative hands-on actions in moving weights along the arms of the lever to balance it were studied by V.V. Rubtsov and L. Martin (Rubtsov et al., 1991). Recently, the examination of joint activity within the same context was continued by A.V. Konokotin (2021) in respect to its potential benefits for the development of learning communication.

The approach to computer support in educational design has been developed within Activity Theory since 1980 by V.V. Rubtsov and his colleagues (Rubtsov, 1996). This approach suggested that the efficacy of digitalization depends on what components of students’ actions will be scaffolded by the computer. Within this approach, the necessity of special modeling actions in forming concepts of complex structure was justified and the provision of appropriate space for learning actions became one of the important missions of computer support (Rubtsov & Ulanovskaya, 2021; Vysotskaya, 1996).

In our research, we focus on model mediation, which plays a central role in concept acquisition (Davydov, 2008; El’konin & Davydov, 1966), and the ways to scaffold it, through use of computers in particular. Modern computer simulations often present digital objects, which primarily prompt students to perform common “trial and error” practical probes, while they search for a way to solve the problem. An example of such an approach for the balance scale task is considered in the study of J. van der Graaf (2020), who described students’ strategies of balancing the lever in cases when they were allowed to check their solutions through the simulation.

However, if we aim to scaffold students’ acquisition of the “learner’s position” and influence the quality of the learning process, supported by the computer, we should consider the mediation of practical trials through special modeling work. The assessment of students’ ability to adopt the required modeling tools may thus be prognostic of the efficacy of their promotion through practical tasks within a digital environment.

Our research goal was to examine the potential of the balance scale problem and the features of its digital simulation, as a means to help students adopt the conceptual way of acting and avoid falling into the trap of the common “trial and error” method that is often prompted by the availability of practical probes.

We find tasks with “scattered” weights (when the weights are distributed over several suspension points) (Siegler, 2013), which prohibit application of simple rules and strategies that students can easily grasp, crucial for assessment purposes. Moreover, we assume students’ work on these tasks to be essential to switch them from “empirical” to “conceptual” consideration of the matter presented through the computer.

### Organization, Procedure, and Methods of Research

The goal of our current research was to examine the ways in which students acquire the concepts of complex (multiplicative) structure due to adoption of special conceptual means, which cannot be “invented” by students spontaneously. We assume that success in solving specially designed diagnostic tasks will be indicative of mastery of these means. Thus, our tasks were: to devise learning materials and appropriate diagnostic procedures to study the efficacy of our approaches to computer-supported teaching on a sample of fifth-grade students and to analyze students’ performance in pre- and post-test tasks (with the same types of problems, but altered numbers of weights).

### Diagnostic Procedure

**Figure 1.**
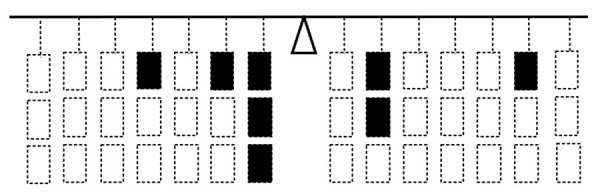
Type I task example: “A student wanted to balance the lever and placed the weights as presented. Will this configuration make the lever balanced?”

We designed a special set of diagnostic tasks in order to assess the initial quality of students’ understanding of equilibrium. Each task presented a picture of a lever with some identical weight units attached. The first type of task (Type I, six problems) required evaluating the balance state of the given weight configuration (*Figure 1*).

a) Yes, the weights are balanced

b) No, the leftside will go down

c) No, the right side will go down

**Figure 2.**
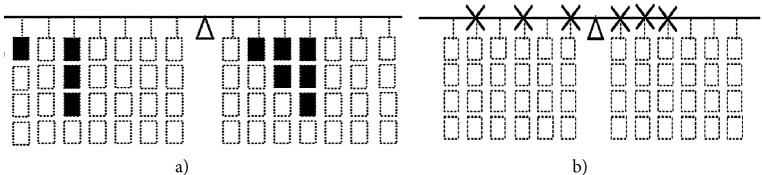
Type II task example: (a) a configuration of weights is provided; move one of them to make it balanced; and (b) balance five weights (the crosses mark places where weights cannot be attached).

The second type of task (Type II, four problems) asked students to find a way to rearrange the given configuration of weights (add, remove or relocate a weight) or to place all given weights on the lever to make it balanced within the restrictions posed by the task (*Figure 2*). Some special conditions were introduced to prevent students from applying simple strategies of balance (e.g., symmetrical positioning of the weights): a different number of weights for opposite sides of the lever, blocked locations for weight placement.

**Figure 3.**
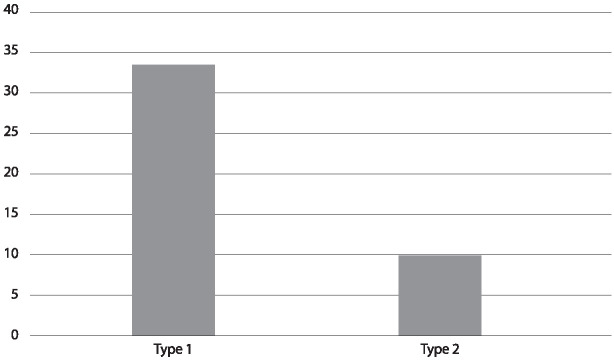
Students’ performance on the pre-test tasks (percentage of correct answers to tasks of each type)

181 fifth-grade students (11–12 years old, average academic achievement, from two Moscow schools) participated in the pre-test. First, a physical lever was demonstrated to the class. The experimenter set up the unbalanced configuration of weights and then showed that it can be balanced by rearranging, removing, or adding weights. Then students received individual fill-in blanks with 10 tasks (6 tasks of the first type, 4 tasks of the second type). The instruction was to consider the presented configurations as having been set up by someone who was trying to reach a balanced state. Students were to make their own guesses about whether the balance was achieved (task Type I) and to suggest corrections needed to balance the lever (task Type II). The percentage of correct answers for the tasks of both types was calculated. The results are presented in *Figure 3*.

The success rate of the first type of task (33.6%) does not differ significantly from the probability of guessing the correct answer among three variants. The second type of task proved to be crucial for assessment: the answers could not be easily guessed; thus, the success rate was very low (9.75%).

The pre-test was conducted to confirm that our participants were not capable of solving balance scale problems on their own, by spontaneous insights.

### Teaching Strategy

**Figure 4.**
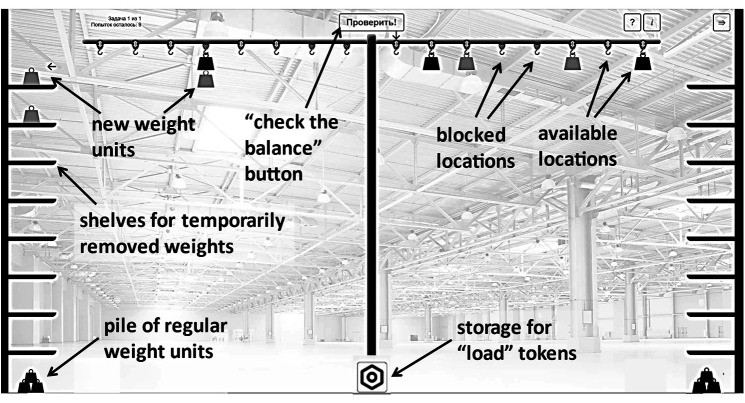
The screen of the digital lever simulation

All students were taught with the help of the digital lever simulation that we designed (Vysotskaya et al., 2023). The digital support ran on the web on a standard browser and was developed using HTML/JavaScript. The materials were deployed on a web-based learning management system, which could also log all the participant’s actions (https://lever.digitar.ru^1^).The computer simulation reproduced the balancing of a lever with several weights and allowed students to change the configuration of weights and see the outcome, as if a real lever was being used (see *Figure 4*). The screen view included the lever with eight locations for a maximum of eight weight units on each arm, shelves for removed weights, unlimited piles of weights (if the conditions of the task allowed their use). The lever was always in a “locked” state, with no immediate reaction to any of the changes performed, until the final configuration of the weights had been settled (according to the task restrictions). A student could either place or remove weight units and check the balance state of the lever (hitting a special button). Part of the operations could be limited depending on the task conditions: certain locations for weights could be locked, some of the weights could be attached to the lever and their rearrangement prohibited, the number of weights could be limited or not, etc. Another varied condition was the presence of different types of weight units with a pre-set ratio of their masses (Vysotskaya, 1996; Vysotskaya, Lobanova, & Yanishevskaya, 2022; Vysotskaya, Lobanova, & Yanishevskaya, 2023).

The tasks that we posed through the digital simulation mostly exploited configurations of scattered weights, as opposed to the common approach to task design where the weights are attached to only one position on each arm at a time (Filion & Sirois, 2021; Konokotin, 2021; Normandeau et al., 1989, and others). The task with several locations for weights is considered to be the most difficult, as no intuitive strategies and rules that students are able to invent themselves will work (Boom et al., 2001; Normandeau et al., 1989; Siegler, 2013) and no “workaround” ways of solving the problems would be of any help. Most children have an idea of the symmetrical placement of weights as the simplest strategy to achieve balance. Moreover, many students have a general notion that a larger weight is balanced by a smaller one placed at a greater distance from the fulcrum. Changes of both parameters (weight and distance) while dealing with several scattered weights simultaneously requires conceptual consideration: students have to evaluate the ratio between them quantitatively. The simple rule (inverse proportion), which students may also refer to, does not help here either, since the weights are placed in an improper way.

We assume that the evaluation of the “load” created by each object on the lever according to its placement and weight, and its contribution to the achievement of equilibrium, is at the core of the concept-mediated action that is necessary for handling the scattered-weights situation. The “load” is the “third magnitude”, which is not as salient as the extensive «first» and «second» magnitudes (the weight or distance) directly presented by the task data. However, the consideration of its “hidden” value is necessary for dealing with the magnitudes involved: it makes it possible to introduce specific modeling tools. The essential role of magnitudes of this kind was highlighted by V.V. Davydov (El’konin & Davydov, 1966) in regard to solving problems which required calculation of coordinated changes of two magnitudes (not rare for primary math curricula). Thus, we focused on introducing the idea of counting the load as the “third magnitude” and developed a special tool for assessment and reassessment of each weight’s contribution to the would-be equilibrium state, which mediates students’ reasoning about balance. Alongside the computer simulation, special counting tokens were introduced to explicate and measure a weight’s contribution to the total load on the leftand on the right and to confirm the equivalence of the load on both sides, despite the obvious difference in the number of weight units and their location^2^. Moving each weight unit has to be reflected by altering the number of tokens used to predict the resulting change of load. In this case, the created load has to be evaluated with tokens: each step (a shift to the next mark on the scale) made by the weight unit towards or away from the fulcrum has to be recorded with an added or removed token (the “making steps” rule). Thus, the “third magnitude” is made tangible and subject to examination, so that students can guide their solution by adjusting their future actions to the equivalence of the load value for both sides of the lever.

**Figure 5.**
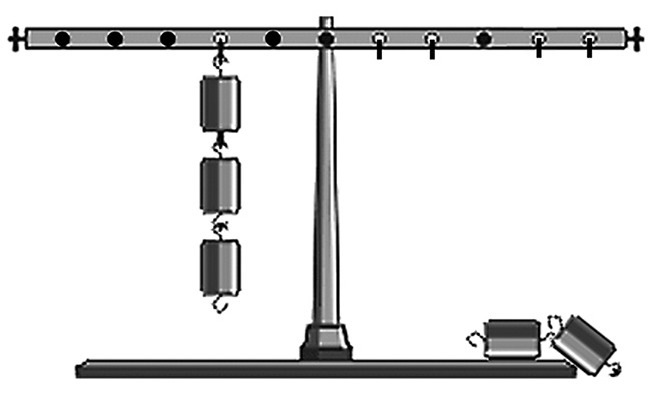
Students were asked to place two weights on the right arm (where the third position has been blocked) in order to balance the three weights on the left arm (placed beforehand by an “imaginary partner”)

The goal of the first series of the experimental teaching instruction (the “explanation and trial” approach) was to test ways of introducing conceptual orientation tools to students’ practical work with the balance-the-lever tasks in a digital environment. The development of the learning situation (problematization) started with balancing the lever with an uneven number of weights provided. An “imaginary partner” has already done the corresponding part of the work by attaching weights to the lever on the left side (*Figure 5*). Now students have to complete the task by attaching the other two weights on the right side of the lever.

The teacher confirmed that students knew how to balance the three weights on the left with the same number of weights on the right, but had only a vague idea of how to balance them with only two weight units. For example, students suggested moving the two weights further from the fulcrum (guessing that this would somehow increase “their weight”), to the third place – but there was no such option in the simulation provided (because of the “blocked” slot). The practical validation of other ideas (to move the two weights even further) brought the students to the conclusion that the task was unsolvable. After the preliminary discussion, the “making steps” rule was demonstrated: the teacher counted steps that moved each of the three weights away from the fulcrum. In this way, six tokens (magnets on the blackboard) to mark each step, increasing the load of each weight, were put on the board. Students were now asked to suggest the placement for each of the two remaining weight units, marking their moves with tokens, in order to obtain the same load as the “imaginary partner” had created by his three weights on the left side. If a particular configuration that a student would suggest, is evaluated as requiring more or fewer than six tokens, students can ascertain that the lever will not be balanced. Equilibrium is achieved, thus, by placing the weight units at the second and fourth positions, or at the first and the fifth, posing the idea that the load is created by each weight independently. The solution of all the tasks which followed each time required the student to assess the placement of all weights and the total load with tokens.

Students then received a series of individual training tasks presented through the digital media described above, and a set of counting tokens for the load evaluation to scaffold their solution. The tasks required that they balance the left arm, which already had some weights on it, by attaching all the given weights to the right arm of the lever. Simple solutions through copying the preset configuration of weights were excluded by providing a different number of weights for the other arm or by locking up some of the positions on it. The tasks required students to determine the configuration of several scattered weights, and the success of their practical trials obviously depended on the use of tokens. After the balance was achieved (by recurring probes with the digital lever), students were asked to draw the right configuration on their handouts and mark the loads on both arms with special tokens according to the preset rule of “making steps” (the fill-in blank for tokens was provided for each task, but students could mark tokens in any convenient form). The last two tasks in the series required balancing the lever in only one attempt. The teacher reminded students to draw all the obtained balanced configurations and mark the corresponding tokens.

The procedure took one teaching session: two school lessons successively for problematization and individual work with the computer series (10 practical tasks). 123 students from our pre-test sample were taught according to this strategy. After a month or so, the post-test was given (the set of 10 tasks similar to those in the pretest).

The other teaching procedure (the “joint work” instruction) involved the remaining 58 students from the surveyed sample and used the activity-oriented approach, which seeks to form concepts purposefully through special arrangement of students’ own actions, specifically joint work within the learning task that reconstructs the material circumstances of the concept origin (Davydov, 2008; Rubtsov, 1996; Solovieva & Quintanar, 2021). It included the same problematization, but differed in the task variation and the organization of students’ substantial interactions in pairs with one PC. Three school lessons were conducted for each group of these students, as additional time was needed for the joint work arrangement. As the digital simulation described above could support two users simultaneously by sharing the operations with weights available between them, we assigned each student to one side of the lever, where he or she could add or remove the weights within the restrictions posed by the tasks. The task variation was also enriched with problems aimed to intensify students’ coordination: instead of a configuration ready-made by an “imaginary partner”, there was an opportunity to change both arms, using a number of weights from a shared stack to balance an empty lever in particular, as well as the requirement to balance new units of unknown weight.

The 58 students who were taught according to the second strategy also participated in a delayed post-test.

## Results and Discussion

**Figure 6.**
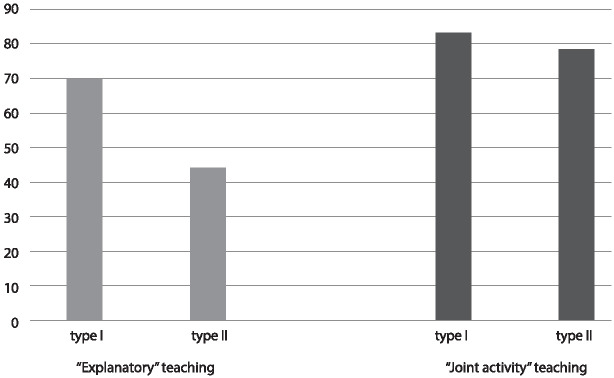
Students’ post-test performance in the two groups taught according to either the “explanation and trial” or “joint activity (substantial interactions)” approach

The results of the post-test (the overall percent of successful tasks solutions) for both groups of students, who were taught according to different strategies, are presented in *Figure 6*.

The post-test diagnostics revealed that students’ progress in critical tasks was dependent on the instructions they received. The diagram shows that the organization of substantial interactions among partners solving one shared task was more effective than the “explanation and trial” instruction. Both approaches, however, implied active approbation of an additional procedure of counting load with tokens. Yet, the difference between students’ performance of critical tasks (Type II) is significant (*p* < 0.05, Mann-Whitney U test). To explain these results, we will refer to the analysis of classroom observations and students’ written works during the experimental teaching.

**Figure 7.**
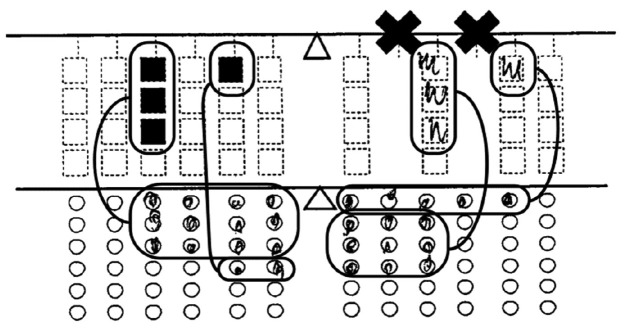
An example of the appropriate token (dots) notation to the balanced distribution of weights

*Note: The curves link weights and their load contributions*

**Figure 8.**
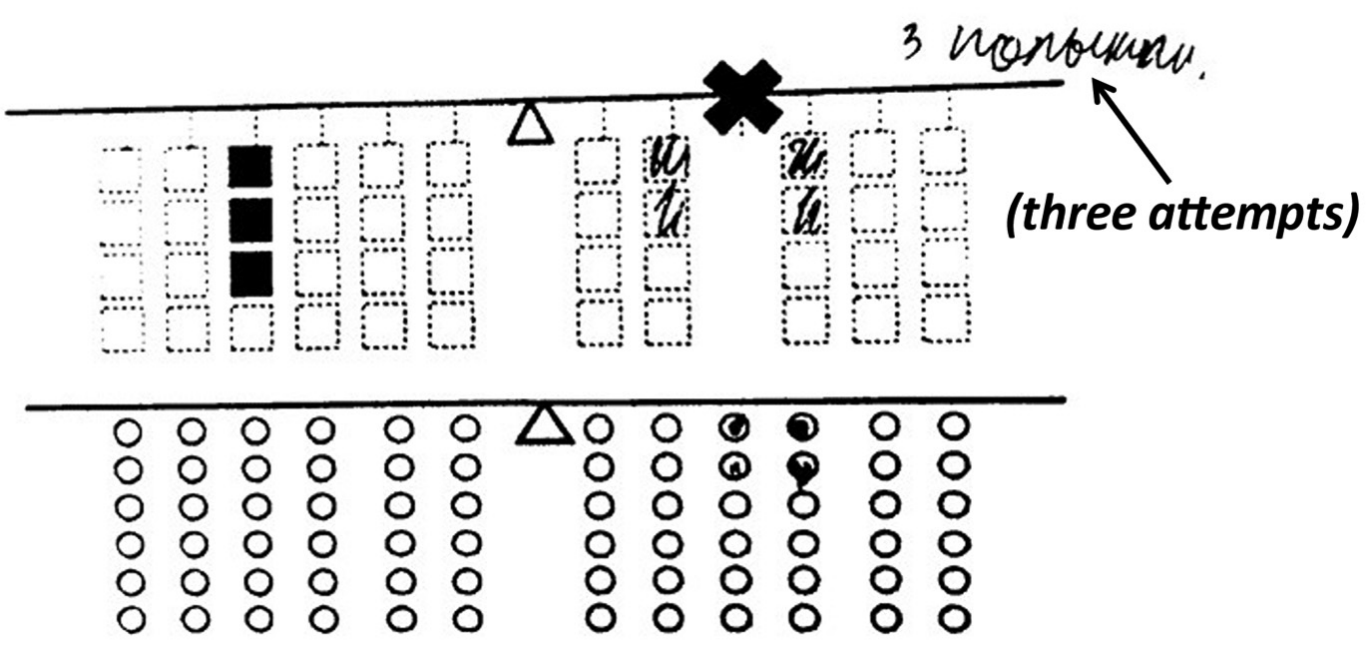
An example of token notation (four dots) that is irrelevant to the balanced distribution of weights, achieved by the student by means of practical trials

*Note: The task was to balance the given configuration of three weights on the left with four weights provided. The student tried to copy the weight units’ location from above with the token notation directly.*

In the first teaching series (the “explanation and trial” instruction), students successfully completed the training series. Their filled-in blanks mostly contained the required answers and corresponding token notation, showing that the load for each weight had been calculated (*Figure 7*). A remarkable feature though, was a considerable number of solutions with correct balanced weight configuration, but an irrelevant token notation (*Figure 8*). We assume that such notations could have been made after the balance was achieved through practical trials within the digital simulation of the lever. Thus, the procedure of load-assessment could be performed “formally” after the teachers’ prescription (by about 30% of students, judging by their written works). Perhaps the students understood that the token notation corresponded to the equilibrium state: there were an equal number of tokens on both sides in some solutions, but this did not match the correct distribution of weights achieved by practical trials. The last two tasks, with only one attempt allowed to balance the lever, were mostly failed by the students: the correct configurations, however, were mostly followed by relevant token notation.

Analysis of the post-test filled-in blanks revealed that the wrong solutions (especially for Type II tasks) were mostly without any token notations, or with inappropriate ones, while the correct solutions coincided with the correct token notation. The results of the first teaching series confirmed that successful solution of complicated tasks, in which testing the balance through trials was disabled, depended on the seemingly troublesome and excessive procedure of marking “steps” of weights with tokens. The initial variant of teaching strategy showed that almost half of our sample learned to estimate, predict, and achieve the balance, referring to the load evaluation. However, it is disturbing that a significant number of students chose not to follow the simple and effective “step” rule and ignored the tokens, and consequently performed poorly. This made us search for and design a special learning situation to scaffold the adoption of the load evaluation procedure by students as their own thinking tool.

Analysis of students’ work throughout the second teaching strategy, which supported students’ joint problem-solving based on the distribution of the possible operations on different sides of the lever, confirmed that students relied on the counting-tokens procedure more than those in the first teaching series. We assume that it is the necessity to change the weights’ position on both sides of the lever simultaneously that made students refer to the load evaluation procedure as the only means to coordinate their joint work, rather than a mere illustration of the obtained equilibrium. The contradiction between the intended relocation of weights, planned by each partner, and the ensuing conflicts, was resolved by the preliminary counting with tokens, which allowed students to evaluate the possible load inflicted by their weights, before they were even placed on the lever, and to agree on their solution beforehand. Thus, the “common” coordinated scheme of load, created by weights configurations on both sides of the lever, served as a sound basis for handling the balance of the lever directly and allowed the students to reassess the task conditions through the lens of each operation’s necessary contribution to the creation of equivalence within the restrictions presented. By adjusting one’s own actions and the actions of one’s partner to achieve equal load on both sides of the lever with an unequal amount of weight units, students managed to succeed in tasks even when the available number of practical trials was limited.

## Conclusion

Our study focused on the general features of students’ adoption of conceptual ways of thinking, which mediate dealing with complicated tasks based on the purposeful transformations of independent magnitudes, contributing to the required change of a dynamic object’s state. Students’ spontaneous attempts to find solutions for such problems lead to a series of rules and strategies (Boom et al., 2001; Filion & Sirois, 2021, Siegler, 2013), developing into an entangled tree of reasoning and gradually crashing over the scattered-weight task. Introduction of hands-on trials with a real or digital lever entertains students, but the conceptual way of handling the matter can hardly be invented by school students through mere trial and error. The torque concept is presented in natural science classes as the simple law of the lever (the multiplication of weight and distance for both sides), but its application is reduced to formula memorization. Studies show that difficulties with multiplicative concepts are still present in adulthood (Siegler, 2013). The researchers referred to the early school years, when students were not yet taught the balance rule and examined their ability to operate with two independent magnitudes simultaneously in solving a practical task and to grasp their contribution to the balance. In these studies, no means for achieving an equilibrium state evaluation were provided. Our design of the appropriate teaching strategy (Vysotskaya et al., 2023) started from the introduction of the “third magnitude” (Davydov, 1966), which coordinates changes of the independent parameters of an object. Thus, we aimed to define the structure of the modeling space, which will allow students to explicate transformations of this latent magnitude, and to embed it in their solutions to practical tasks.

However, even the direct demonstration (the first part of our study) of the adequate and effective means to build up the coordination between adding and moving weights, which could help to solve even the trickiest problems, is not enough to convince a considerable number of students to adopt them. Such students, who chose to rely on hands-on probes rather than on the procedure of load evaluation and viewed counting of tokens as a purely formal act, makes us pay special attention to the content of actions that mediate the adoption of this knowledge as new ways of thinking. The second part of our study slightly clarifies the mechanism of assimilation of conceptual ways of thinking as actual mediators of handling the matter through the introduction of special restrictions to the tasks, among which the most essential was the organization of students’ interactions based on the distribution of operations with the weights and the necessity to make the final decision: “The configuration is ready! Let’s try it!”. The functions of the load evaluation procedure shift from additional “illustrative” ones to essential “predictive” ones, towards the integrated result (the balance state) of the independent changes (weights’ rearrangement) performed by the partners.

The significant gap in the post-test results of the two teaching series (especially in critical tasks) highlighted the difference in acquisition of the modeling tools and proves the need to design software that will establish for students the problem of the approbation and application of conceptual modeling tools as the only way to deal with the challenge of joint work. Recent research reviews (Belolutskaya, 2023, Benavides-Varela, 2020; Qureshi et al., 2021, and others) convince us that the issue of effectiveness as depending on digitalization of learning is urgent. We assume that an approach to computer support design, based on the preliminary analyses of the students’ actions and on explication of their conceptual mediation, may be productive in terms of problem-solving and concept change.

The principles that we relied upon to design a learning situation that will scaffold students’ adoption of the provided modeling tools according to their actual role as mediator of problem-solving, may contribute to the design of relevant educational mediation, digital mediation in particular. The purposeful changing of the model object, performed by a student explicitly, is to be considered as the initial form of action, responsible for concept acquisition. In regard to the balance task, it is the examination of ways to regain equilibrium for a lever within a variety of task conditions. The organization of joint learning activity requires a substantial distribution of the possible operations with the object, which poses the necessity to coordinate the model representation of the partial changes planned by the partners in order to achieve an integrated result. The formation of the concept and of the conceptual mediation for comprehension of the changes performed upon the object, therefore, should be based on the introduction of symbolic means to present the partial actions of the partners and their contribution to the general result within a special modeling space.

## Limitations

A delayed post-test of students’ model mediation quality was conducted only for part of our sample; the pilot results showed that students’ later performance in balance tasks did not differ from their immediate results. However, we plan to conduct the delayed post-test for all our participants to assess the retention of the acquired concepts and their possible transition to related topics.

The integration of the designed module into the regular physics curriculum would be of interest, but was left out of this paper’s scope. The influence of the multiplicative concepts formed within the equilibrium module on the transformation of the related physics’ content is also to be examined in future studies.

## Footnotes

1 A demo version of the tasks is available here: https://lever.digitar.ru/game/sn9tj8q7ai15

2 The tokens could be either magnets on the blackboard, or marbles, or drawings, or special digital tokens, available in the computer simulation (*Figure 4*).
